# Hypergravity exposure during gestation modifies the TCRβ repertoire of newborn mice

**DOI:** 10.1038/srep09318

**Published:** 2015-03-20

**Authors:** Stéphanie Ghislin, Nassima Ouzren-Zarhloul, Sandra Kaminski, Jean-Pol Frippiat

**Affiliations:** 1 EA7300, Stress Immunity Pathogens Laboratory, Faculty of Medicine, Lorraine University, F-54500 Vandœuvre-lès-Nancy, France

## Abstract

During spaceflight, organisms are subjected to mechanical force changes (gravity (G) changes) that affect the immune system. However, gravitational effects on lymphopoiesis have rarely been studied. Consequently, we investigated whether the TCRβ repertoire, created by V(D)J recombination during T lymphopoiesis, is affected by hypergravity exposure during murine development. To address this question, C57BL/6j mice were mated in a centrifuge so that embryonic development, birth and TCRβ rearrangements occurred at 2G. Pups were sacrificed at birth, and their thymus used to quantify transcripts coding for factors required for V(D)J recombination and T lymphopoiesis. We also created cDNA mini-libraries of TCRβ transcripts to study the impact of hypergravity on TCRβ diversity. Our data show that hypergravity exposure increases the transcription of TCRβ chains, and of genes whose products are involved in TCR signaling, and affects the V(D)J recombination process. We also observed that ~85% of the TCRβ repertoire is different between hypergravity and control pups. These data indicate that changing a mechanical force (the gravity) during ontogeny will likely affect host immunity because properties of loops constituting TCR antigen-binding sites are modified in hypergravity newborns. The spectrum of peptides recognized by TCR will therefore likely be different.

The specific immune response is based on the existence of B and T lymphocytes expressing antigen-specific receptors known as B- and T-cell receptors (BCR and TCR, respectively). These receptors are not encoded in the germline DNA but are generated *de novo* during B and T lymphopoiesis. The production of these receptors occurs *via* Recombination-Activating Gene (RAG) 1- and RAG2-mediated assembly of functional genes from individual variable (V), diversity (D) and joining (J) gene segments. Briefly, RAG proteins bind to recombination signal sequences located adjacent to each V, D and J gene segment and introduce DNA double strand breaks. The neighboring coding DNA is converted to a hairpin during breakage. Broken ends are then processed and joined with the help of several factors involved in DNA repair. Two of these factors are particularly interesting because they increase the diversity of antigen-binding sites at the level of the third complementarity determining region (CDR3) of TCR heavy chains. These factors are Artemis which can create palindromes when it opens DNA hairpins and the terminal deoxynucleotidyl transferase (Tdt) which add nucleotides (N-nucleotides) at open extremities of coding segments before their joining^1^. This assembling procedure, called V(D)J recombination, takes place at specific stages of B and T cell differentiation and is essential for lymphocyte differentiation and the generation of diverse BCR and TCR repertoires indispensable for protection against pathogens.

During spaceflight, organisms are subjected to gravitational changes (hypergravity during launch and landing, microgravity during the mission) that affect the immune system and can compromise defenses against infections^2^. Until now, studies regarding spaceflight-induced immune dysfunction have focused mainly on innate immunity and T-cell responses, while lymphopoiesis, despite its essential functions (the synthesis of B and T cells and of antigen-specific receptors), has barely been investigated. Furthermore, no information is currently available concerning the impact of mechanical forces on the V(D)J recombination process and the diversity of the TCR repertoire.

Concerning B lymphopoiesis, we have recently shown that the transcription of IgM heavy chains and of the Ikaros lymphoid-determining transcription factor are modified when embryos of the urodele amphibian *Pleurodeles waltl* develop under altered gravity conditions. These data suggest that gravitational changes can modify B lymphopoiesis^3^. This hypothesis was recently confirmed using hind limb unloaded mice, a ground-based model of microgravity, where a decrease in B lymphopoiesis was observed as of the common lymphoid progenitor stage with a major block at the pro-B to pre-B cell transition^4^. Furthermore, we showed that a long-term spaceflight affects the use of immunoglobulin heavy chain variable gene segments (VH) subgroups^5^ and the expression of individual VH gene segments^6^ in response to an antigenic stimulation in adult *P. waltl*, thereby suggesting that these conditions could affect the V(D)J recombination machinery.

T lymphopoiesis is also affected by simulated microgravity because reductions in CD4^+^, CD8^+^ and CD4^+^CD8^+^ thymocyte populations were reported when murine fetal thymuses were cultured under clinorotation^7^. Indeed, it has been shown that microgravity blocks T cell development at the immature single positive (ISP) stage, which is after the CD4^−^CD8^−^ double negative (DN) but before the CD4^+^CD8^+^ double positive (DP) stage. These observations reflect alterations at the pre-TCR complex. These alterations in pre-TCR signaling led to decreased expression of the IL-7 receptor involved in thymocyte survival through the DN to DP transition^8^,^9^. Interestingly, Woods and colleagues^9^ showed that remaining DP cells expressed higher levels of CD3 while more immature populations, such as DN cells, expressed lower levels compared to 1G controls. These data suggest that microgravity might not completely prevent β-selection (known to be controlled by the pre-TCR complex), which occurs at late stages of DN transition, but that its impact might be sufficient to prevent a full transition to the DP stage.

Because β-selection and T lymphopoiesis were shown to be affected by microgravity exposure, we wondered whether the TCRβ repertoire created by V(D)J recombination is also affected by gravity change. Hypergravity was chosen for this study because it is easy to implement. We decided to work on pups conceived and born at 2G to ensure that the creation of the TCRβ repertoire occurred exclusively under hypergravity conditions, thereby eliminating the potential effects of a previous 1G exposition as T lymphopoiesis starts *in utero*. We quantified several transcripts encoding factors required for V(D)J recombination, T lymphopoiesis and TCR signaling from the thymuses of hypergravity and control pups. We also created cDNA mini-libraries of TCRβ transcripts to study the impact of hypergravity on the diversity of TCRβ chains. Our results confirm that gravity changes affect T lymphopoiesis and interestingly demonstrate that hypergravity exposure during embryonic and fetal development modifies the TCRβ repertoire of newborns thereby likely impacting host immunity.

## Results

### Sizes, body and lymphoid organ masses of newborns

**Figure 1:**
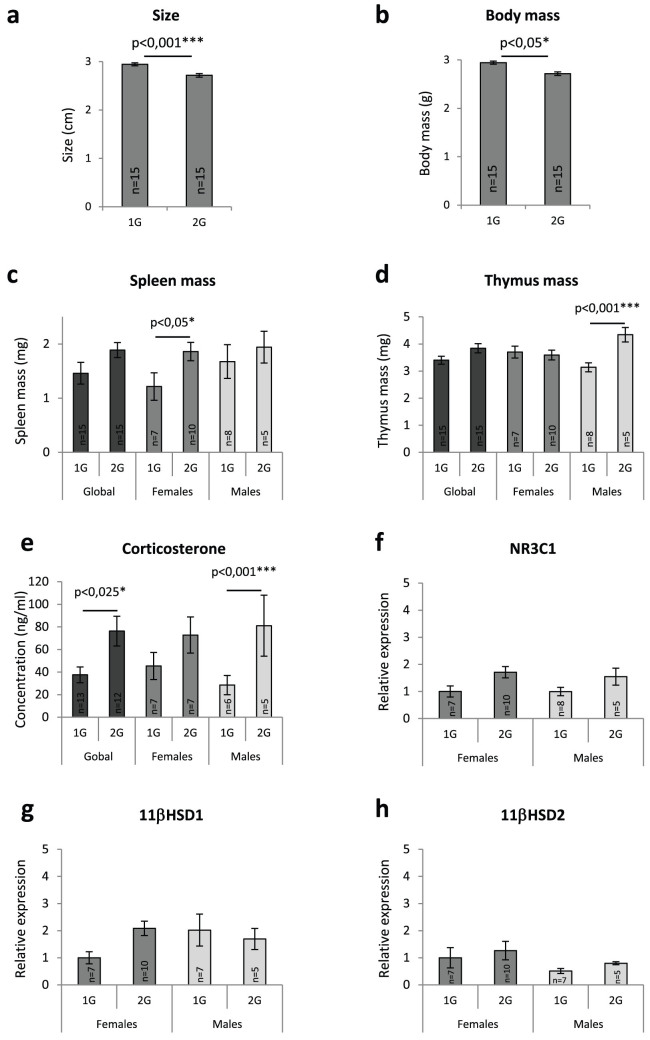
Morphological parameters, lymphoid organ masses and evaluation of stress in newborn mice. (a) Size, (b) body mass, (c) spleen mass and (d) thymus mass of pups. (e) Serum corticosterone concentrations determined by ELISA. (f–h) Evaluation of stress in newborns thymus by qRT-PCR quantification of NR3C1, 11βHSD1 and 11βHSD2 mRNAs encoding respectively the glucocorticoid receptor, 11β-hydroxysteroid dehydrogenase type 1 and 11β-hydroxysteroid dehydrogenase type 2, two enzymes involved in corticosterone activation/deactivation. mRNA levels were normalized to four housekeeping transcripts. The relative value obtained with 1G females was set to 1. Asterisks indicate statistically significant differences. Error bars reflect standard error of the mean.

Following birth, pups conceived and born at 1 or 2G were sized, weighed and sacrificed. As shown in Figure 1a–d, hypergravity pups were smaller, lighter and seemed to have globally higher spleen and thymus masses than controls. DNA was then extracted from tails to determine newborn gender. These analyses revealed 5 males and 10 females from the three 2G litters and 8 males and 7 females from the two control (1G) litters. When lymphoid organ masses were analyzed according to gender, we noted an increase in spleen mass in hypergravity females (Figure 1c) and an increase in thymic mass in hypergravity males (Figure 1d). These data indicate that hypergravity affects fetus and lymphoid organs and that effects are gender dependent. Consequently, males and females were separated in all subsequent analyses.

### Stress in newborns

Corticosterone, the most studied stress hormone in rodents, was quantified to evaluate stress in newborn mice. As shown in Figure 1e, hypergravity induced increased corticosterone concentrations in the serum of 2G pups that were significant in males but not in females. These amounts of corticosterone were close to the one observed in unstressed mice (~50 ng/mL according to the literature) indicating that pup stress was minor. Because thymuses are too small at birth to allow the direct quantification of corticosterone, we evaluated the stress response in this tissue by quantifying NR3C1, 11βHSD1 and 11βHSD2 mRNAs encoding respectively the glucocorticoid receptor, 11β-hydroxysteroid dehydrogenase type 1 and 11β-hydroxysteroid dehydrogenase type 2, two enzymes involved in corticosterone activation/deactivation (Figure 1f–h). The amounts of these three transcripts were not statistically modified in 2G pups (p values > 0.05) indicating that glucocorticoids' effects are minor in 2G thymuses.

### T lymphopoiesis in newborns

**Figure 2:**
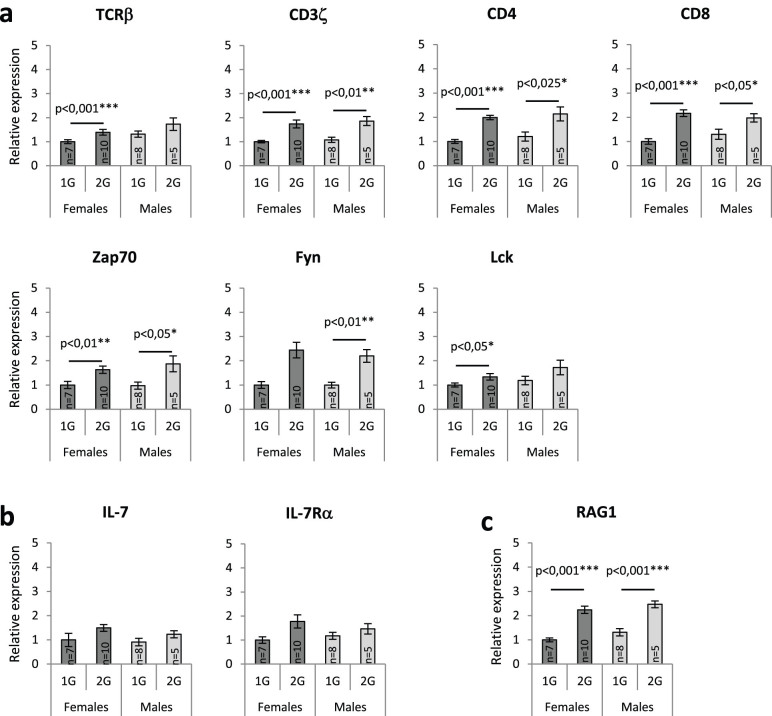
Quantification of transcripts encoding (a) TCRβ chains and proteins (CD3ζ, CD4, CD8, Fyn, Lck and Zap70) involved in TCR signaling, (b) IL-7 and IL-7Rα (the alpha chain of the IL-7 receptor) and (c) RAG1, a mandatory protein for V(D)J recombination. mRNA levels were normalized to four housekeeping transcripts (means ± SEM). The relative value obtained with 1G females was set to 1. Asterisks indicate statistically significant differences. Error bars reflect standard error of the mean.

Because previous studies have shown that microgravity hinders murine T cell development^7^,^9^, we quantified transcripts encoding TCRβ chains, an essential T cell marker expressed during T lymphopoiesis from the pre-T up to the mature stage, and mRNA encoding proteins (CD3ζ, CD4, CD8, Fyn, Lck and Zap70) involved in TCR signaling and indispensable for T cell maturation and activation. We observed a statistically significant 1.4-fold increase in TCRβ mRNA expression in 2G females when compared with the 1G females (Figure 2a). The level of TCRβ mRNA was not significantly different in 2G males, but the average amount appeared higher at 2G. mRNAs encoding molecules implicated in TCR signaling were also more abundant in the thymus of 2G pups. We observed a 1.7-fold increase of CD3ζ transcripts in both 2G males and females, 1.8- and 2-fold increases of CD4 mRNAs, 1.5 and 2.2-fold increases of CD8 mRNAs and 1.6- and 1.9-fold increases of Zap70 mRNAs in 2G males and females, respectively. Fyn mRNAs were upregulated in 2G males (2.2-fold). The same trend was observed in 2G females but did not appear to be statistically significant. The opposite situation was observed concerning Lck transcripts that were statistically significantly increased in 2G females (1.3-fold) but not in 2G males, even if the average amount of Lck mRNA was higher.

Beside TCR signaling, IL-7 signaling is also required for T cell development^10^. Interestingly, the expression of IL-7 and IL-7Rα (the alpha chain of the IL-7 receptor) mRNAs were not significantly increased in 2G samples (Figure 2b).

Together, these data show that hypergravity increased the transcription of TCRβ chains and of genes whose products are involved in TCR signaling, but not of those initiating IL-7 signaling, suggesting that hypergravity affects T lymphopoiesis in both genders *via* TCR signaling compounds.

### Effectors involved in V(D)J recombination

Because hypergravity affected the transcription of TCR and associated signaling molecules, we wondered whether the V(D)J recombination process, required for the generation of TCR, is modified. To address this question, we first quantified mRNAs coding for RAG1, a major effector of this recombination machinery^11^. Our qPCR analyses revealed a statistically significant increase in RAG1 transcript in 2G males and females (2.4- and 2.9-fold increases, respectively) suggesting that this mechanism could be affected by hypergravity exposure (Figure 2c). As mentioned above, Artemis and Tdt participate to V(D)J recombination and are essential for creating diversity in TCR CDR3 regions. Thus, to complete the RAG1 transcriptional study, we evaluated the activities of Artemis and Tdt by analyzing palindromes and N-nucleotides added by these two proteins in CDR3 regions of TCRβ chains.

We noted that palindromes were absent in most sequences (90.28% and 80.00% of 1G and 2G sequences, respectively) (Supplementary Table S1 online). However, TCRβ CDR3 sequences with 2 or 4 nucleotide palindromes were, respectively, 1.7- and 2.9-times more abundant in the 2G group than in the 1G group. The number of palindromes at V-D junctions, which are created at the DN3 stage, was similar in 1G and 2G pups. However, the number of palindromes was increased in 2G D-J junctions created at the DN2 stage. These results indicate that the location in which Artemis cleaves the DNA hairpin to allow D-J joints at the DN2 stage might be more frequently altered in the thymus of 2G pups.

**Figure 3:**
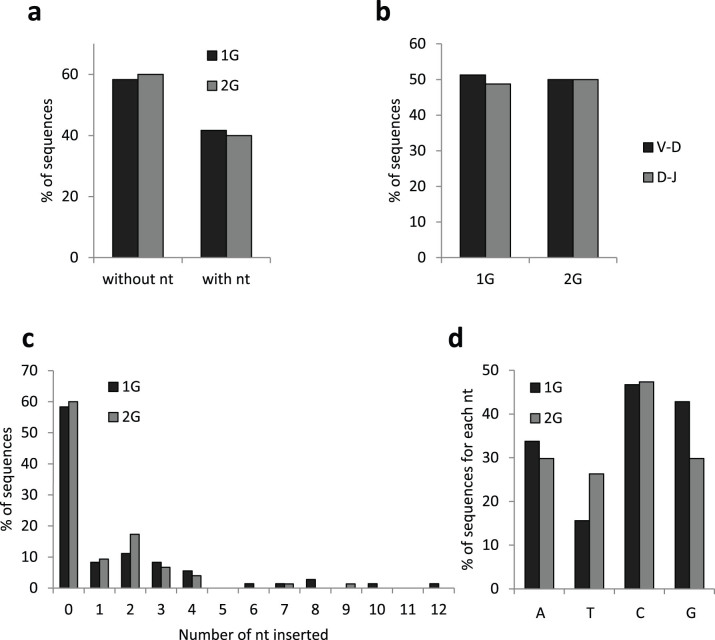
Impact of hypergravity exposure during gestation on N-nucleotide addition. (a) Percentages of TCRβ CDR3 sequences with or without inserted nucleotides. (b) Percentages of CDR3 sequences containing added nucleotides at V-D and D-J junctions. (c) Distribution of sequences according to the number of added nucleotides. (d) Nature of the added nucleotide. These data are deduced from the analysis of 151 cDNA sequences.

The number of N-nucleotides added by Tdt at V-D and D-J junctions was then determined. Our analyses show that hypergravity did not alter the percentages of sequences with or without inserted nucleotides (Figure 3a), the frequency of added nucleotides at V-D and D-J junctions (Figure 3b) and the number of inserted nucleotides (Figure 3c). However, hypergravity decreased the addition of G bases and increased the addition of T bases (Figure 3d) indicating a change in base preference for this enzyme.

These results indicate that the activities of the Tdt and Artemis proteins were modified, at least partially, in the thymus of 2G pups. Combined with changes in RAG1 mRNA expression, these data indicate that the V(D)J recombination process, that assemble V, D and J gene segments during T cell development, is likely affected by hypergravity exposure during gestation.

### TCRβ gene segments usage

To ensure that the V(D)J recombination process is modified in 2G samples, we determined how V, D and J gene segments were used to create expressed TCRβ genes which are the products of the V(D)J recombination. For that purpose, we constructed TCRβ cDNA mini-libraries from the thymuses of 2G and control newborns. Males and females samples were pooled to create these libraries because our qPCR results indicated that hypergravity-induced transcriptional changes (Figure 2) were similar in both genders. We sequenced and analyzed 77 clones randomly chosen from the 2G mini-library and 74 clones from the 1G mini-library.

**Figure 4:**
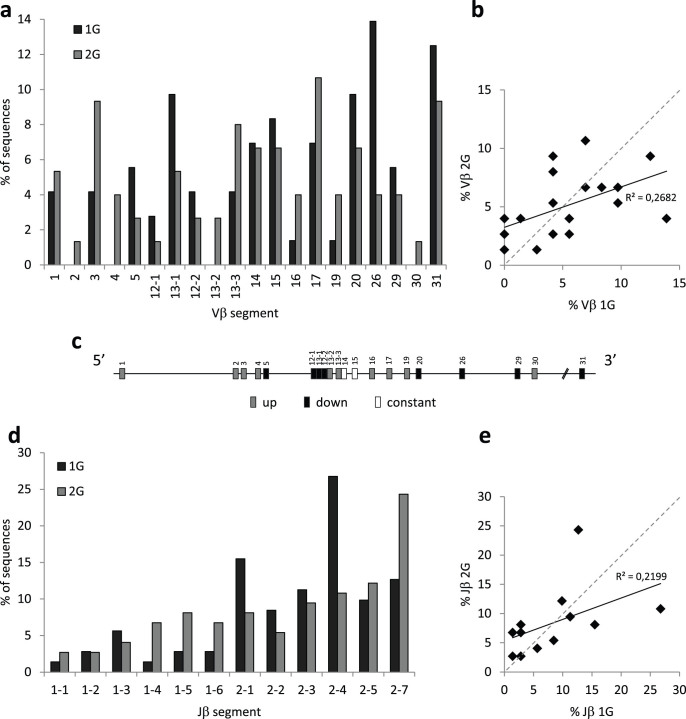
Effects of hypergravity on TCRβ gene segment usage. TCRβ cDNA mini-libraries were created from the thymuses of hypergravity and control newborn mice. Seventy-seven clones randomly chosen from the hypergravity mini-library and 74 clones from the 1G mini-library were sequenced and analyzed. (a) Vβ gene segment usage in TCRβ transcripts of control (1G) and hypergravity (2G) pups. (b) Frequencies of each Vβ segment observed at 2G plotted against those observed at 1G. (c) Schematic representation of the TCRβ locus on murine chromosome 6 on which expression changes are summarized. (d) Jβ gene segment usage in TCRβ transcripts of control and hypergravity pups. (e) Frequencies of each Jβ segment observed at 2G plotted against those observed at 1G.

Only two D gene segments exist in the murine genome. Both were found in our 1G and 2G sequences, and they were utilized to the same extent in both groups. However, comparative analysis of 1G and 2G sequences highlighted modifications in Vβ gene segments utilization (Figure 4). Indeed, we noticed that the Vβ1, Vβ2, Vβ3, Vβ4, Vβ13-2, Vβ13-3, Vβ16, Vβ17, Vβ19 and Vβ30 segments were more frequently used and that the Vβ5, Vβ12-1, Vβ12-2, Vβ13-1, Vβ20, Vβ26, Vβ29 and Vβ31 segments were less frequently used at 2G (Figure 4a). When Vβ utilization frequencies at 2G were plotted against those determined at 1G, the obtained linear regression generated an r^2^ of 0.2682 (Figure 4b) confirming that Vβ gene segment usage was different between the 1G and 2G groups. As for Vβ, we observed changes in Jβ segment usage at 2G. Indeed, the Jβ1-1, Jβ1-4, Jβ1-5, Jβ1-6 and Jβ2-7 segments were more frequently expressed, and the Jβ2-1 and Jβ2-4 segments were less frequently expressed at 2G (Figure 4d). The linear regression obtained when we plotted Jβ frequencies at 2G *vs.* those observed at 1G generated an r^2^ of 0.2199, demonstrating that Jβ gene segment usage was also different at 2G (Figure 4e). Finally, we analyzed the combinations of V, D and J gene segments in each of our 151 cDNAs (Table 1). This study revealed that ~85% of the VDJ associations observed in TCRβ transcripts were different in 2G pups by comparison to control pups conceived and born at 1G.

Taken together, our data show that the V(D)J recombination process is affected by hypergravity exposure during embryonic and fetal development. The consequences being a profound change in the TCRβ repertoire as ~85% of the VDJ associations, in expressed TCRβ transcripts, were different in 2G thymuses.

### TCRβ CDRs analysis

**Figure 5:**
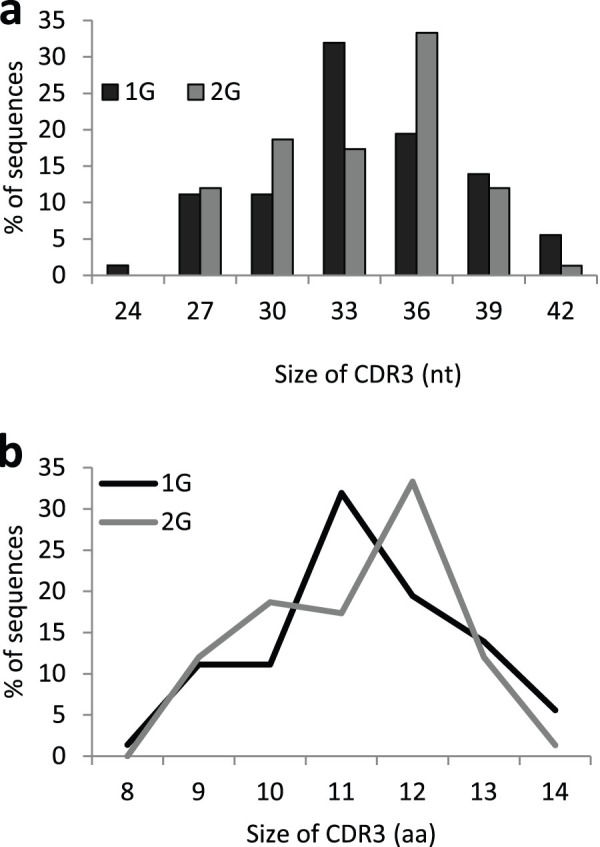
CDR3 lengths in the two groups of newborn mice. Repartition of TCRβ sequences according to CDR3 length expressed in nucleotides (a) or amino acids (b). These data show that the TCRβ CDR3 loop is on average one amino acid longer in 2G pups.

To evaluate the consequences of this important modification of the TCRβ repertoire, we studied complementarity determining regions. Indeed, CDRs are the most variable parts of TCR V regions and are essential for MHC/peptide recognition. Six CDRs (CDR1, 2, 3 of the heavy chain and CDR1, 2, 3 of the light chain) encode loops that collectively come into contact with the MHC/peptide complex. The heavy chain CDR3 is the longest and most variable CDR because it comprises the 3′-end of the V, the entirety of the D and the 5′-end of the J segments that are randomly assembled during V(D)J recombination. We first noted a shift in CDR3 average length from 33 nucleotides (nt) at 1G to 36 nt at 2G (Figure 5a). Consequently, the TCRβ CDR3 loop of the antigen-binding site is, on average, one amino acid longer in the 2G group (Figure 5b). This first analysis showing that the length of TCRβ CDR3 is increased in hypergravity pups, we then focused on the biochemical characteristics of the three antigen-binding site loops encoded by TCRβ CDRs.

**Figure 6:**
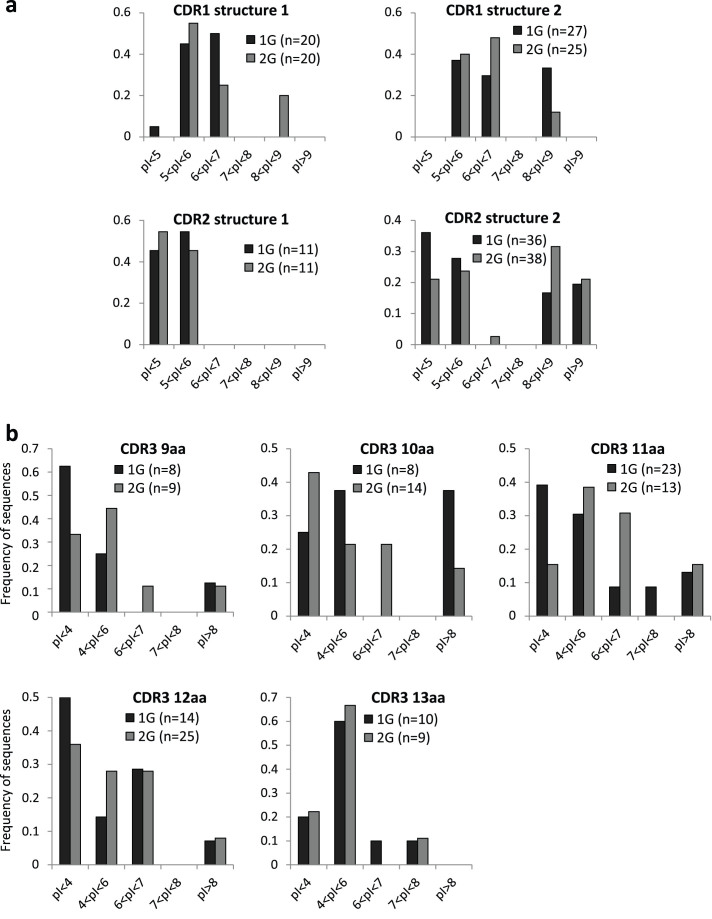
Biochemical characteristics of TCRβ CDRs. (a) Isoelectric points (pI) of CDR1 and CDR2 regions classified according to their predicted structures. (b) Isoelectric points of CDR3 regions classified according to their size.

We determined the isoelectric points (pI) of CDR1, CDR2 and CDR3 predicted amino acid (aa) sequences. CDR1 and CDR2 were classified according to their predicted structures as defined by Al-Lazikani and colleagues^12^. CDR3 sequences were categorized according to size because structures are difficult to predict in these highly variable regions. As shown in Figure 6, modifications of pI were observed in sequences of 2G pups. Indeed, the first possible CDR1 structure and the second possible CDR2 structure presented an increased global pI at 2G while the second possible CDR1 structure presented a lower global pI at 2G. Concerning CDR3, it appeared that loops of 9, 11 and 12 aa presented a higher global pI at 2G while loops of 10 aa present a lower global pI. These modifications of pI are due to modifications in loop amino acid composition at 2G (see Supplementary Fig. S1 online), especially in the middle of CDR3 loops. These data show that hypergravity modifies the biochemical properties of the loops composing TCR-binding sites. The spectrum of peptides recognized by TCR could therefore vary, impacting host immunity.

## Discussion

To study the effects of hypergravity exposure during murine development on the TCRβ repertoire, we produced three litters conceived and born at 2G and two control litters. These 5 litters were produced at different dates by different parental pairs. Our statistical analyses did not reveal differences among the three 2G litters or among the two 1G litters for the parameters tested in this study.

We first noted that 2G pups were smaller and lighter than controls (Figure 1a–b). These observations are in agreement with previous data. Indeed, lower birth weights have been reported in hypergravity-raised rats^13^,^14^. Reduced pup weight could be explained by the higher energy expenditure induced in mothers by the hypergravity environment. Additionally, the production of hormones linked to energy metabolism and the distribution of energy supplies were reported to be sensitive to gravity change^13^,^15^.

Hypergravity increased the amount of corticosterone in the serum of 2G pups (Figure 1e) but concentrations remained close to the average concentration (50 ng/mL) observed in unstressed mice indicating that pup stress was not important. This is not surprising because they experienced only one gravity level during their development. Thus, they most likely perceived hypergravity as a natural situation. Furthermore, parents were centrifuged for at least 3 weeks at 2G before the beginning of the experiment to habituate them to centrifugation and reduce stress during the experimental phase (see Methods section).

Several studies have shown that spaceflight conditions frequently reduce spleen mass in adult rats and mice^16^,^17^. The results in regard to thymus mass are more variable because thymus mass has been reported to decrease^16^, increase^18^ or remain unchanged compared to controls^19^,^20^,^21^. Observed reductions in spleen and thymus mass are often explained by a stress response^22^,^23^. Here, we observed that hypergravity increased the spleen mass of 2G females and the thymus mass of 2G males (Figure 1c–d). These data indicate that gender, in addition to stress, must be taken into account to explain variations in lymphoid organs weights after exposure to gravity changes. Indeed, it was shown that elevated levels of corticosterone or estrogen induce thymic atrophy^24^,^25^. We performed LC-mass spectrometry studies to quantify estrogens in newborn sera, but in all cases, estrogen levels were below the detection threshold. Consequently, we quantified transcripts coding for the estrogen receptor and noted that ERα mRNAs were two times more abundant in the thymic tissue of 2G pups (See Supplementary Fig. S2 online). This observation could explain why lymphoid organ weights are increased in 2G males. Indeed, it has been shown that deletion of ERα led to hypoplasia of thymus and spleen and that ERα, but not ERβ, is mandatory for spleen and thymus development in males, whereas ERβ is required for estradiol-mediated thymic atrophy in females^26^. Stress responses are less likely explanations because NR3C1, 11βHSD1 and 11βHSD2 mRNA levels were not statistically modified in 2G thymuses (Figure 1f–h). In addition, the amount of thymic glucocorticoid receptor mRNA (Figure 1f) was not decreased in response to the increased serum corticosterone concentration, as could have been predicted from the literature^27^.

### Hypergravity increases the transcription of genes required for T lymphopoiesis

As microgravity has been shown to impair T cell development^7^,^9^, we examined the expression of 10 mRNAs coding for proteins indispensable for T cell maturation (Figure 2). Globally, all transcripts were increased in the thymus of 2G pups, except those coding for IL-7 and IL-7Rα, suggesting that hypergravity affects mainly TCR expression and signaling. The fact that IL-7 transcription is not affected at 2G is in agreement with Lebsack and colleagues^28^ who did not observe modifications in IL-7 expression in the thymus of space-flown mice. Woods and colleagues^9^ noted that simulated microgravity decreased the expression of IL-7Rα in the murine thymus. Here, IL-7Rα transcript expression was not different in the thymuses of 2G pups. Similarly, in another study, we showed that hind limb unloading, which simulates microgravity, decreased early B cell differentiation and that this reduction is not due to a decrease in IL-7Rα expression on murine pro-B cells^4^.

### Hypergravity exposure during murine development modifies the TCRβ repertoire of newborns

Increased RAG1 transcription levels (Figure 2c) suggest that the V(D)J recombination process might be affected in the thymuses of hypergravity pups. To investigate this possibility, we analyzed expressed recombined TCRβ genes from cDNA mini-libraries constructed from the thymuses of hypergravity and control newborns. Out of 22 functional Vβ, 2 functional Dβ and 13 functional Jβ segments existing in the murine genome^29^, 20 Vβ, 2 Dβ and 12 Jβ were found in the 151 sequences analyzed here. Eighty to ninety percent of these sequences did not contain palindromes and 60% did not contain N-nucleotide additions at V-D or D-J junctions. Similarly, Happ and Palmer^30^ observed the expression of 18 Vβ and Feeney^31^ the expression of 2 Dβ and 12 Jβ in thymic sequences of newborn mice. In the last study, N-nucleotide additions and palindromes were detected in 33% and 11% of TCRβ junctional sequences, respectively. Similarly, Cherrier and colleagues^32^ estimated that only 24% of Vβ-Dβ-Jβ junctions contain N-nucleotide additions ≥ 2 nucleotides. These values are close to those observed here, indicating that most TCRβ gene segments are expressed at birth but that junctional diversity (due to palindromes and N-nucleotide additions) is reduced in 1G and 2G pups, thereby generating less diverse repertoires than in adult mice.

Analysis of palindromes and N-nucleotides at TCRβ V-D and D-J junctions revealed differences in Artemis and Tdt activities in the thymus of 2G pups. Combined with RAG1 transcriptional changes, these data indicate that the V(D)J recombination process is modified in 2G thymuses. This provides a first explanation of why comparison of Vβ and Jβ gene segment frequencies between 1G and 2G pups (Figure 4) and the analysis of VDJ associations (Table 1) revealed different gene segment usages and associations in the thymus of 2G pups. Modification of the V(D)J recombination machinery could also explain our previous data showing that the expression of immunoglobulin VH subgroups and individual VH gene segments were modified when adult *P. waltl* were immunized onboard the Mir space station^5^,^6^. A second explanation could rely on epigenetics. Indeed, it was shown that the selection of Jβ gene segments for V(D)J recombination is governed by chromatin conformation^33^ and that epigenetic changes control recombinase activity^34^. Interestingly, Vβ gene segments whose expression is modified in the thymuses of 2G pups are located in the same areas of the TCRβ locus (Figure 4c), suggesting local modifications in chromatin structure. The different usage of TCRβ V and J gene segments in 2G pups could therefore result from hypergravity-induced epigenetic changes. This hypothesis is supported by the statistically significant increase in transcripts coding for the DNMT3b DNA methyltransferase in the thymuses of 2G females (see Supplementary Fig. S2 online) and previous studies showing that microgravity induces global DNA-methylation and histone H3 acetylation modifications^35^.

We also observed changes in CDR1, 2 and 3 characteristics and CDR3 length in 2G newborns. On average, CDR3 regions are one amino acid longer in the TCRβ chains of 2G pups. CDR3 loops constitute the most variable region of the TCRs and have been shown to interact with an antigen peptide buried in the peptide-binding groove of the MHC molecule, thereby playing a crucial role in antigen recognition. On the other hand, TCR CDR1 and CDR2 loops seem to have been evolutionary selected for MHC reactivity^36^,^37^. Thus, the first consequence of changing TCR CDR3 characteristics and length is that the spectrum of peptides recognized by TCRs of 2G pups will likely be different. Indeed, Goyarts and colleagues^38^ showed that extensive changes in the TCR recognition pattern might be induced by small perturbations in the CDR3. The second consequence is that positive selection might be affected. Indeed, it was shown that positive selection is more efficient in *Tdt*^–/–^ mice where a higher proportion of thymocytes make the transition from immature DP to mature SP thymocytes suggesting that TCRs with shorter CDR3s are more suitable for positive selection^39^. This suggestion was later confirmed by the observation that shorter CDR3β sequences are positively selected and that longer CDR3β are negatively selected in the thymus^40^. Furthermore, the hypothesis that positive selection could be affected in the thymus of 2G pups is supported by the increased expression of 7 transcripts coding for proteins involved in TCR signaling. Finally, it was shown that the TCR repertoire is more polyreactive and less peptide specific when CDR3s are shorter^41^. Increasing the size of CDR3 might therefore affect these properties.

In conclusion, this study shows that hypergravity exposure during murine embryonic and fetal development increases the transcription of TCRβ chains and of genes whose products are involved in TCR signaling. Furthermore, we also show that the V(D)J recombination process is affected by hypergravity exposure during gestation. This leads to a profound change in the TCR repertoire as ~85% of TCRβ transcripts were different in 2G thymuses. Thus, changing a mechanical force (here the gravitational force) during ontogeny might impact host immunity through modifications of the biochemical properties of loops constituting TCR-binding sites.

## Methods

### Animals

Mice used in this study were pairs of C57BL/6j provided by Charles River (Bois des Oncins, France). Before centrifugation exposure, animals were housed in standard cages with food and water *ad libitum* in a quiet room with constant temperature (22°C), 50% relative humidity and 12-h light/dark cycles (dark period 20 h00-08 h00). All adult mice were first centrifuged for at least 3 weeks at 2G to habituate them to centrifugation and reduce stress during the experimental phase. Females were multiparous to reduce the risks of improper care of the pups. For prenatal exposure to 2G, a pair of mice was centrifuged so that conception and delivery occurred in the centrifuge. Birth was detected by embedded video. The centrifuged group consisted of 15 pups from 3 different litters. Control pups were conceived and born in standard conditions in the same room as the centrifuged mice. The control group consisted of 15 pups from 2 different litters. Experimental procedures were conducted in accordance with the National Legislation and the European Directive 86/609/EEC on the protection of animals used for experimental purposes. Moreover, the protocol was approved by the local ethics committee (authorization number 75-1641).

### Centrifugation

Standard cages containing mouse pairs were placed in gondolas of a large-radius centrifuge^42^ designed for producing low noise and equipped with antivibration devices. The centrifuge was set to a rotational speed of 29.6 rotations per minute to produce a gravity level of 2G at the center of the gondolas. This G level was chosen because it does not induce stress in adult mice^42^. In our hands, rotational speeds equal to or greater than 3G blocked pregnancy. This observation is in agreement with a previous study showing that mice cannot reproduce at 3.5G^43^. Adult mice were supplied enough food and water for three weeks so that the centrifuge was operating continuously. Mice were left undisturbed during the three weeks of chronic centrifugation. Infra-red video allowed remote day and night monitoring of mice. The centrifuge was stopped as soon as one female gave birth. Pups were sized, weighed, sacrificed by decapitation and blood, spleen, tail and thymus were collected. All environmental variables, except the gravity level, were the same as in standard housing. Given the centrifuge diameter and rotational speed, the Coriolis force supported by an adult mouse running at 30 cm/s was < 1% of the inertial force, *i.e.,* less than the gradient of the G level in the cage. Pups were less subjected to the Coriolis force because they were mostly immobile. Cages containing control mice were placed in the same gondolas and in the same room as centrifuged mice but in a static position (1G) to ensure that they were exposed to the same general conditions such as noise, humidity and temperature. After birth, control pups were treated as 2G pups.

### Genotyping

To determine the gender of each pup, genomic DNA was extracted from the tail using the Genomic DNA extraction kit (Biosentec, Auzeville-Tolosane, France). This DNA was used to amplify the *Foxl2* gene present in male and female genomes and the *Sry* gene specific of the male genome. PCR was performed using DNA (100–200 ng), *Taq* Polymerase (0.6 units) (Thermoscientific, Villebon sur Yvette, France) and specific primers (each 0.7 µM) (see Supplementary Table S2 online). PCR products were run on a 1.5% agarose gel to identify males (*Foxl2*^+^ *Sry*^+^) and females (*Foxl2*^+^ *Sry*^−^).

### Quantitative RT-PCR (qRT-PCR)

Total RNA was extracted from the thymus of each newborn using the RNeasy kit (Qiagen, Courtaboeuf, France) and reverse transcribed using random primers, RNAout and MML-V reverse transcriptase (Invitrogen, Cergy Pontoise, France) following the manufacturer's instructions. qRT-PCR were performed using the MESA Fast SYBRGreen I qPCR Master Mix (Eurogentec, Angers, France) and a Mastercycler® realplex^2^ real-time PCR machine (Eppendorf, Hamburg, Germany). The cycling protocol was as follows: 3 min at 95°C, followed by 40 cycles of 15 s at 95°C and 30 s at the annealing temperature indicated in Supplementary Table S2 online. Each qPCR was performed in duplicate and repeated at least two times. Data analysis and relative expressions by comparison to 4 housekeeping transcripts (Eef2, Eif3f, Ppia, and Rpl13A) were performed as previously described^3^. Primers used to amplify 11βHSD1, 11βHSD2, CD3ζ, CD4, CD8, Fyn, IL-7, IL7-Rα, Lck, NR3C1, Rag1 and Zap70 transcripts were purchased from Qiagen (Courtaboeuf, France). Primers used to amplify TCRβ and housekeeping transcripts (see Supplementary Table S2 online) were targeted to different exons to ensure that they could not hybridize to potential traces of genomic DNA. Their specificity was checked using a BLAST search through the U.S. National Center for Biotechnology Information (Bethesda, MD, USA).

### Construction and analysis of TCRβ cDNA mini-libraries

VDJ rearrangements contained in TCRβ heavy chain mRNAs were amplified by 5′-RACE PCR using the SMARTer^TM^ RACE cDNA amplification kit (Clontech, Palo Alto, CA, USA). Briefly, total RNA (200 ng) from the thymus of hypergravity (n = 15) or control (n = 15) pups were pooled and the cDNA synthetized from 200 ng of this mixture according the protocol of the SMARTer^TM^ RACE cDNA amplification kit. VDJ rearrangements were then amplified by two successive PCR reactions performed using Advantage 2 *Taq* DNA polymerase (Clontech, Palo Alto, CA, USA). The first PCR was performed using a gene-specific primer, GSP1 (Supplementary Table S2 online), which anneals to the first constant domain of TCRβ transcripts and the UPM primer provided in the kit. The second reaction was performed using the first PCR product as template, a nested gene-specific primer, GSP2 (Supplementary Table S2 online), which anneals upstream of GSP1 in the first constant domain of TCRβ transcripts, and the nested NUP primer provided in the kit. This second PCR generated products of 0.6 kb containing VDJ rearrangements associated with Cβ1 that were cloned into the pGEM-T® Easy vector (Promega, Madison, WI, USA) to create two mini-libraries: one from the thymus of hypergravity pups and one from the thymus of control pups. Seventy-seven clones were randomly chosen and sequenced from the library constructed from hypergravity pups and 74 from the library constructed from control pups. Sequencing data were analyzed using the IMGT/HighV-quest software^44^.

### Corticosterone quantification

Newborn blood samples were collected during decapitation, allowed to clot at ambient temperature for 15 min, centrifuged at 4°C and 4000 rpm for 20 min to collect serum samples that were stored at −20°C until analysis. Corticosterone was quantified in duplicate using a commercial ELISA kit (Arbor Assays, Ann Arbor, MI, USA). Corticosterone concentrations were calculated from a standard curve and expressed as ng/mL.

### Statistics

Homogeneity of variance was determined using the Levene test and the normality of distribution was determined using the Kolmogorov-Smirnov test. When homogenous variances and distributions were observed, two-way ANOVA analyses were performed. When the variance and distribution were not homogeneous, Mann-Whitney nonparametric tests were performed. P-values <0.05 indicate significance. All results are shown as the means ± standard error of the mean (SEM).

## Supplementary Material

Supplementary Information Supplementary Information

## Figures and Tables

**Table 1:** VDJ associations in TCRβ transcripts of control (black) and hypergravity (underlined bold) pups. Rearrangements without D segments (observed only in the 1G group) are indicated in *italic*. Values indicate the number of time an association has been observed. This table shows that ~85% of the TCRβ repertoire is different between the 1G and 2G groups

	D1						D1	D2	D1	D1	D2	D1	D2	D1	D2	D1	D2
	J1-1	J1-2	J1-3	J1-4	J1-5	J1-6	J2-1		J2-2	J2-3		J2-4		J2-5		J2-7	
**V 1**					1/**1**		1	1	**1**						**1**		
**V 2**							**1**										
**V 3**						**2**	1	**1**		1			1				**4**
**V 4**					**1**	**1**									**1**		
**V 5**									1			2				**1**	1/**1**
**V 12-1**											1		1		**1**		
**V 12-2**		1			**1**			1									1/**1**
**V 13-1**			**1**		**1**	**1**		1				*2*	3			*1*	**1**
**V 13-2**																**1**	**1**
**V 13-3**				**1**			2					2	1/**1**		**1**		**1**
**V 14**					**1**		**1**		1/**1**		**1**	1	**1**		3		
**V 15**			1/**1**	**1**	**1**		1				1		1/**1**		1	**1**	1
**V 16**	**1**		**1**										**1**	1			
**V 17**		**1**							**2**	**2**	**1**	1/**1**	2	1	**1**		1
**V 19**				**1**						**1**						1	**1**
**V 20**	**1**	**1**		1					4			1		**1**		*1*/**1**	**1**
**V 26**	1		2		1	**1**	1/**1**				3			**1**		1	
**V 29**	1			**1**		1					2	2					
**V 30**				**1**													
**V 31**		1	1			1	1/**1**	1/**1**			1	1	**1**	1	**1**	**2**	1
