# Supplementary material for: Hypergravity exposure during gestation modifies the TCRβ repertoire of newborn mice
Source: Sci Rep. 2015 Mar 20;5:9318. doi: 10.1038/srep09318 (PMC5380131; doi:10.1038/srep09318)
Supplement: Supplementary Information [file srep09318-s1.pdf]

Supplementary Information for

Hypergravity exposure during gestation modifies the TCRβ repertoire of newborn mice

Stéphanie Ghislin[1†], Nassima Ouzren-Zarhloul[1†], Sandra Kaminski[1] and Jean-Pol Frippiat[1*]

[1]EA7300, Stress Immunity Pathogens Laboratory, Faculty of Medicine, Lorraine University, F-54500 Vandœuvre-lès-Nancy, France.

[†]These authors contributed equally to this study.

*Correspondence: JPF (jean-pol.frippiat@univ-lorraine.fr)

Figure S1

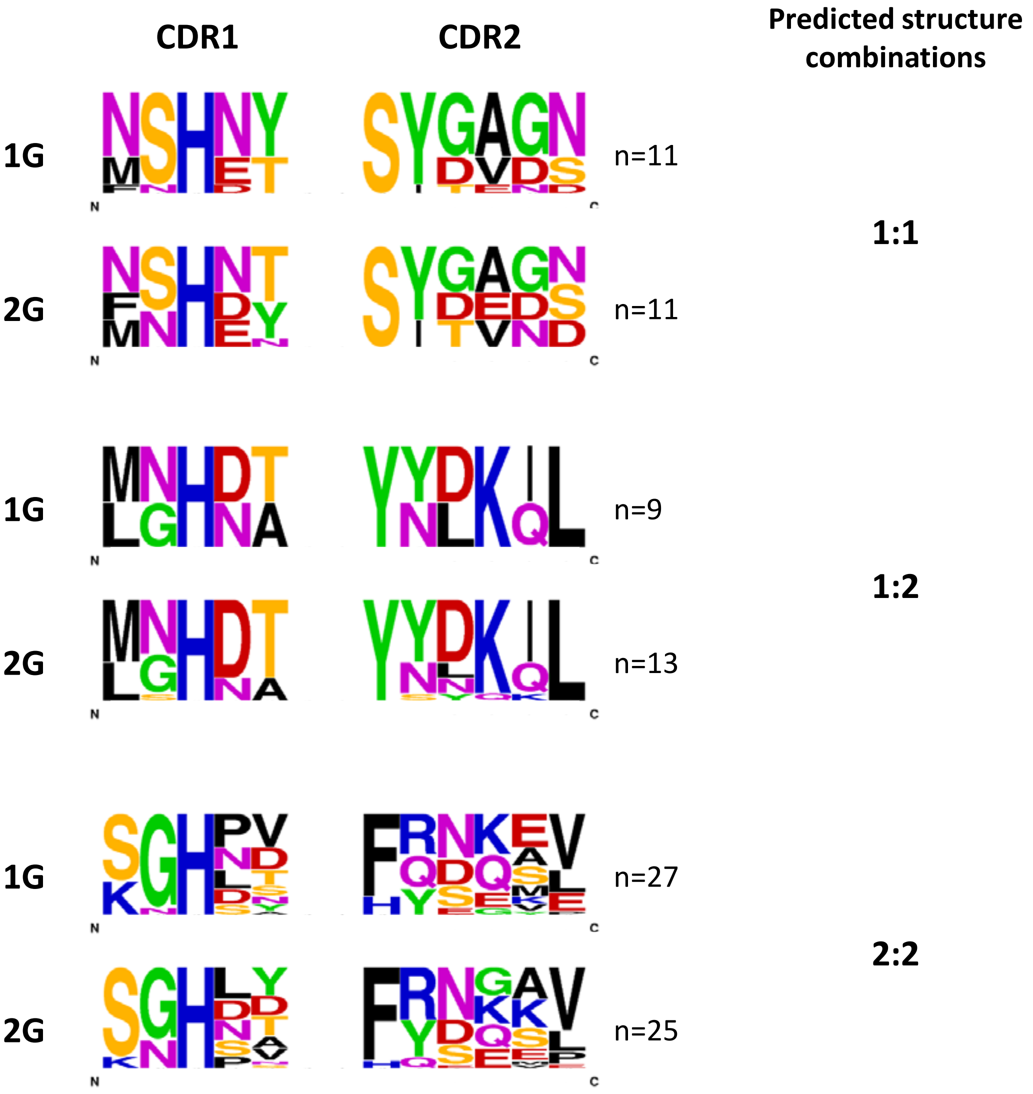
CDR1
CDR2
Predicted structure combinations
1G
n=11
2G
n=11
1:1
1G
n=9
2G
n=13
1:2
1G
n=27
2G
n=25
2:2

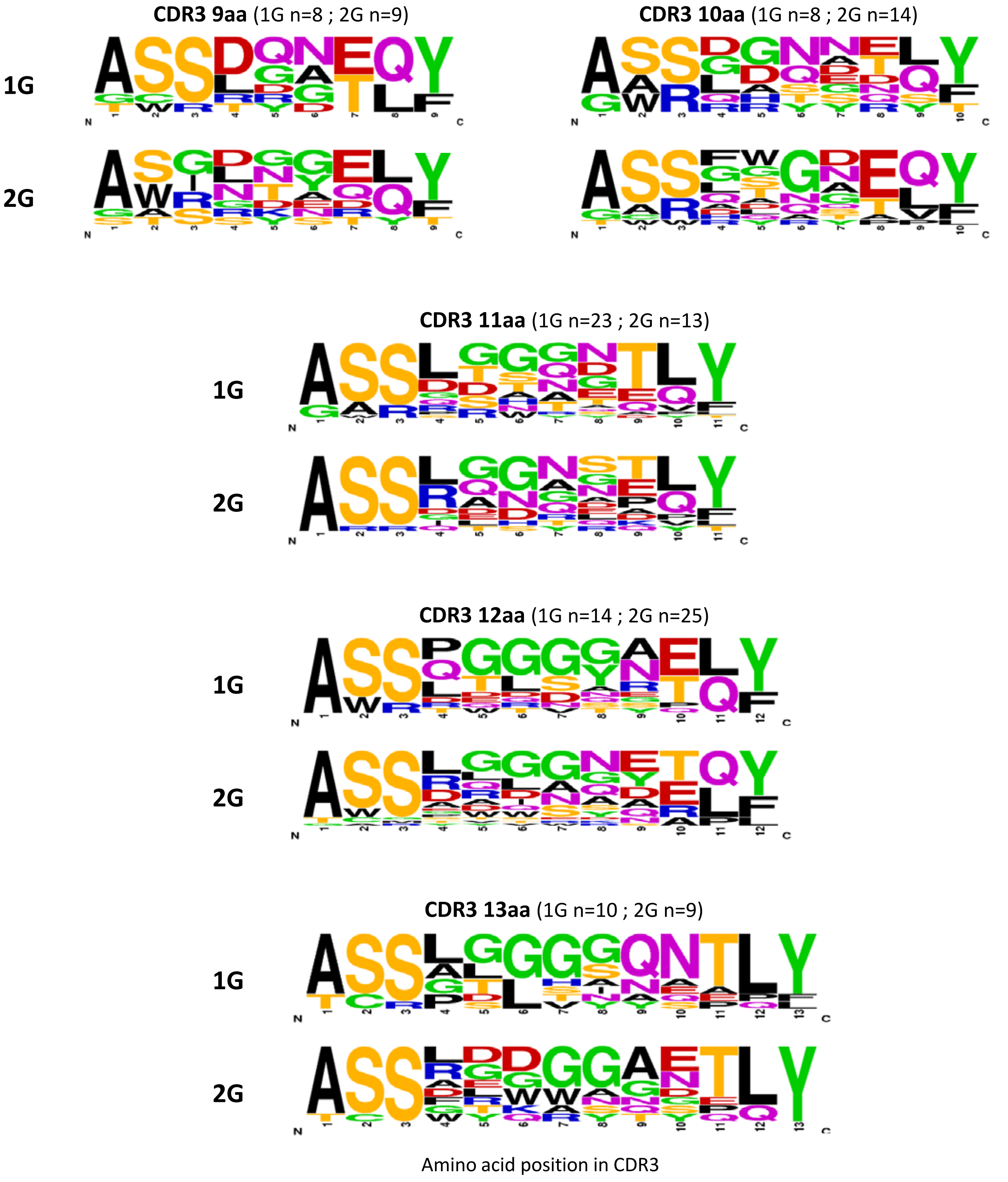

CDR3 9aa (1G n=8 ; 2G n=9)
CDR3 10aa (1G n=8 ; 2G n=14)
1G
2G
CDR3 11aa (1G n=23 ; 2G n=13)
1G
2G
CDR3 12aa (1G n=14 ; 2G n=25)
1G
2G
CDR3 13aa (1G n=10 ; 2G n=9)
1G
2G
Amino acid position in CDR3

Figure S1. Amino acid frequencies at each position in TCRβ loops encoded by CDR1, 2 and 3. The height of each letter indicates the relative frequency of the corresponding amino acid at that position. Loops encoded by CDR1 and 2 have been classified according to the three possible combinations of predicted structures. Loops encoded by CDR3 have been classified according to their size. Amino acids were grouped into six categories: polar GYC (green), polar amide group QN (purple), polar alcohol group ST (yellow), basic KRH (blue), acidic DE (red) and hydrophobic AVLIPWFM (black). This figure was constructed using Weblogo (Crooks, G. E., Hon, G., Chandonia, J.-M. & Brenner, S. E. WebLogo: a sequence logo generator. Genome Res. 14, 1188–1190 (2004)).

Figure S2

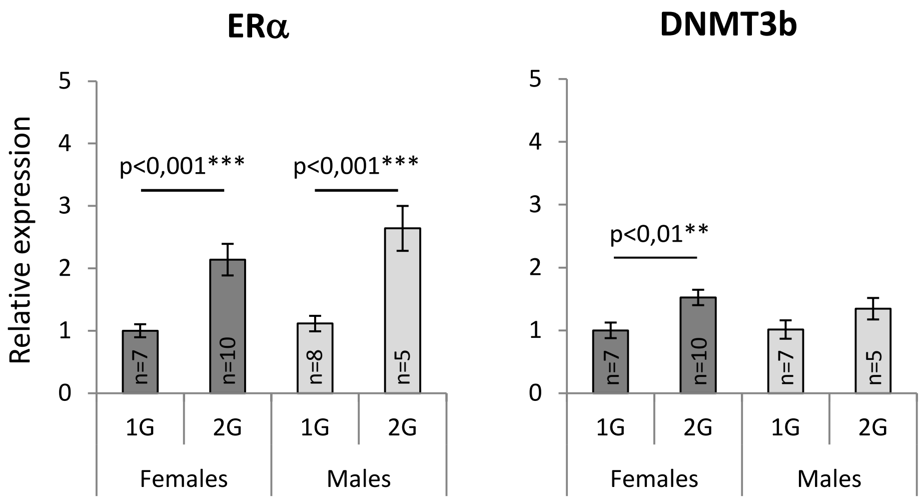

Figure S2. Quantification of ERα and DNMT3b mRNAs by qRT-PCR. mRNA levels were normalized to four housekeeping transcripts. The relative value obtained with 1G females was set to 1. Asterisks indicate statistically significant differences. Error bars reflect standard error of the mean.

Table S1.

A. Lengths and frequencies of palindromes in TCRβ CDR3 regions.

Palindrome size	% of sequences	
	**1G**	**2G**
**0 nt**	90.28	80.00
**2 nt**	6.94	12.00
**4 nt**	2.78	8.00

B. Numbers of palindromes at V-D and D-J junctions.

Palindrome size	Number of palindromes in			
	**V-D junctions**		**D-J junctions**	
	**1G**	**2G**	**1G**	**2G**
**2 nt**	4	5	1	4
**4 nt**	0	1	2	5

Table S2. Primers used in this study. F, forward. R, reverse.

Gene name	Nucleotide sequence	Annealing T°C
DNMT3b	F : 5’-CAGGAGATGGAGACAGAGAT-3’	60
	R : 5’- AAGCTGGAGGTCCCATTGCT-3’	
Eef2	F: 5’-GTGGTGGACTGTGTGTCTGG- 3’	58
	R: 5’ -CGCTGGAAGGTCTGGTAGAG- 3’	
Eif3f	F: 5’ -CATCAAGGCCTATGTCAGCA- 3’	61
	R: 5’ -GTGGTGGGACTGTGTGTCTGG- 3’	
ERα	F:5’- TTGCTGGCTACGTCAAGTCG-3’	61
	F: 5’- CAGAGACTTCAAGGTGCTGG-3’	
Foxl2	F: 5’ -GGCGAGCGCAAGGGCAACTA - 3’	63
	R: 5’ -TGGCAGGAGGCGTAGGGCAT- 3’	
Ppia	F: 5’ -GTCTCCTTCGAGCTGTTTGC- 3’	58
	R: 5’ -GCGTGTAAAGTCACCACCCT- 3’	
Rpl13A	F: 5’ -GGAAGCGGATGAATACCAAC- 3’	61
	R: 5’ -CTTGTCATAGGGTGGAGGGA- 3’	
Sry	F: 5’-AGCCTGCAGTTGCCTCAACA- 3’	63
	R: 5’ -GTGGGGATATCGACAGGCTGCAA- 3’	
TCRβ	F: 5’-TGACCACGTGGAGCTGAGCT-3’	62.6
	R: 5’-CAGAAGGTAGCAGAGACCCT-3’	
**5’-RACE**		
GSP1	5’-CCCACTGTGGACCTCCTTGCCATTCACC-3’	68
GSP2	5’-CACGTGGTCAGGGAAGAAGCCCCTGGCC-3’	68
